# Take Control: A randomized trial evaluating the efficacy and safety of self‐ versus physician‐managed titration of insulin glargine 300 U/mL in patients with uncontrolled type 2 diabetes

**DOI:** 10.1111/dom.13697

**Published:** 2019-04-30

**Authors:** David Russell‐Jones, Arnaud Dauchy, Elías Delgado, George Dimitriadis, Hans A. Frandsen, Luiza Popescu, Bernd Schultes, Krzysztof Strojek, Mireille Bonnemaire, Aude Roborel de Climens, Melanie Davies

**Affiliations:** ^1^ Department of Diabetes and Endocrinology Royal Surrey County Hospital Guildford UK; ^2^ Global Diabetes Division Sanofi Paris France; ^3^ Department of Medicine University of Oviedo Spain; ^4^ Endocrinology and Nutrition Service Hospital Universitario Central de Asturias Oviedo Spain; ^5^ Metabolism Unit Instituto de Investigación Sanitaria del Principado de Asturias (ISPA) Oviedo Spain; ^6^ National and Kapodistrian University of Athens Medical School Attikon University Hospital Athens Greece; ^7^ Department of Internal Medicine Amager Hospital Copenhagen Denmark; ^8^ Global R&D Operations Sanofi, Bucharest Romania; ^9^ eSwiss Medical and Surgical Center, Department of Internal Medicine, Endocrinology Diabetes and Metabolism, St Gallen Switzerland; ^10^ Department of Internal Diseases Diabetology and Cardiometabolic Diseases SMDZ, Zabrze, Silesian Medical University Katowice Poland; ^11^ Clinical Outcome Generation Sanofi Lyon France; ^12^ Diabetes Research Centre, University of Leicester University Hospitals of Leicester Leicester UK

**Keywords:** glycaemic control, hypoglycaemia, randomized trial, type 2 diabetes

## Abstract

**Aim:**

To compare the efficacy and safety of self‐ versus physician‐managed titration of insulin glargine 300 U/mL (Gla‐300) in people with inadequately controlled type 2 diabetes.

**Methods:**

Take Control (EudraCT number: 2015‐001626‐42) was a 24‐week, multi‐national, open‐label, controlled, two‐arm, parallel‐group study in insulin‐naïve and pre‐treated participants, randomized 1:1 to a self‐ or physician‐managed titration of Gla‐300. The fasting self‐monitored plasma glucose (SMPG) target was 4.4 to 7.2 mmol/L. The primary outcome was non‐inferiority of glycated haemoglobin (HbA1c) change from baseline to week 24. Secondary outcomes included SMPG target achievement without hypoglycaemia, hypoglycaemia incidence, adverse events and participant‐reported outcomes (PROs).

**Results:**

At week 24, the least squares (LS) mean HbA1c reduction was 0.97% (10.6 mmol/mol) and 0.84% (9.2 mmol/mol) in the self‐ and physician‐managed groups, respectively, with an LS mean difference of −0.13% [95% confidence interval −0.2619 to −0.0004] (–1.4 mmol/mol [–2.863 to –0.004]), demonstrating non‐inferiority (*P* < 0.0001) and superiority (*P* = 0.0247) of self‐ versus physician‐managed titration. Significantly more of the self‐ than physician‐managed group achieved SMPG target without hypoglycaemia (67% vs 58%; *P* = 0.0187). Overall, hypoglycaemia incidence was similar in each group. No safety concerns were reported. In both groups, similar PRO improvements were observed for distress related to diabetes disease burden and for confidence in diabetes self‐management, with even more individuals achieving a clinically relevant reduction in emotional burden and fewer individuals with high emotional burden in the self‐managed group.

**Conclusions:**

Self‐managed titration of Gla‐300 was superior to physician‐managed titration in terms of HbA1c reduction, accompanied by similar total PRO scores, with a clinically relevant reduction in emotional burden, and similar hypoglycaemia frequency.

## 1 INTRODUCTION

Achieving glycated haemoglobin (HbA1c) targets reduces the risk of long‐term complications of diabetes,^1^ but in real‐world practice many individuals with type 2 diabetes (T2DM) fail to achieve adequate glycaemic control.^2^, ^3^, ^4^ Poor glycaemic control can be a result of clinical inertia, which may be contributed to by several physician‐ and patient‐related barriers including fear of hypoglycaemia and/or weight gain, burdensome treatment regimens, poor persistence to injectable therapies, lack of time for healthcare professionals to teach and difficulty for patients to understand the importance and performance of appropriate titration, frustration at time taken to achieve goals, insufficient communication between healthcare professionals and patients, and anxiety.^5^, ^6^, ^7^, ^8^, ^9^, ^10^ As the disease progresses, lack of glycaemic control may also indicate the need to initiate adjunctive therapies.^11^

Empowering those with T2DM to take a more active role in their own treatment may help individuals to achieve their glycaemic goals and reduce the risk of all‐cause mortality,^12^, ^13^ which could reduce lifetime costs of complications and hospital admissions,^14^ and might help to address the increasing burden of diabetes on healthcare systems worldwide. This concept of patient‐centred care and self‐management is a key focus of the current consensus report from the American Diabetes Association (ADA) and the European Association for the Study of Diabetes (EASD).^15^

Self‐managed titration of insulin glargine 100 U/mL (Gla‐100) may be more effective than physician‐managed titration^16^, ^17^ without any safety concerns,^18^, ^19^ but it does appear to be associated with a higher risk of symptomatic hypoglycaemia compared with physician‐managed titration.^17^, ^18^ This limitation of Gla‐100 could be overcome through self‐titration with insulin glargine 300 U/mL (Gla‐300), a second‐generation basal insulin (BI) with prolonged and more stable pharmacokinetic and pharmacodynamic profiles than Gla‐100.^20^, ^21^ Phase III treat‐to‐target studies demonstrated a lower risk of hypoglycaemia with Gla‐300 versus Gla‐100 in people with T2DM, especially during the titration phase.^22^ This lower risk of hypoglycaemia has also been observed from real‐world evidence of people switching to Gla‐300 versus other BIs.^23^

The present study examined the effectiveness and safety of self‐ versus physician‐managed titration of Gla‐300, in order to evaluate whether the improved glycaemic profile and lower hypoglycaemic risk of Gla‐300 over Gla‐100 make it suitable for a simple, self‐managed titration approach.

## 2 MATERIALS AND METHODS

### 2.1 Trial design and participants

Take Control (EudraCT Number: 2015‐001626‐42) was a 24‐week, multicentre, multinational, 1:1 ratio randomized, open‐label, controlled, two‐arm parallel‐group study, comparing Gla‐300 treatment using a self‐ versus physician‐managed titration algorithm (Figure S1).

Outpatient participants were recruited in 10 European countries, between February and November 2016. Inclusion criteria comprised age ≥18 years, T2DM diagnosis for at least 1 year before screening and at least 6 months on treatment with at least one non‐insulin antihyperglycaemic drug, with or without a BI. Insulin‐pretreated participants were required to have been on a stable dose (type of insulin and time/frequency of injection) within 3 months prior to screening. The main exclusion criteria included HbA1c <7.0% or >10.0% (<53 mmol/mol or >86 mmol/mol) for participants taking BI, or <7.5% or >11.0% (<58 mmol/mol or >97 mmol/mol) for insulin‐naïve participants, and unwillingness to self‐manage the titration algorithm. The full inclusion and exclusion criteria are presented in Table S1.

Background non‐insulin antihyperglycaemic drug(s), including sulphonylureas (SUs), administered at a stable dose for ≥12 weeks prior to screening were permitted according to local labelling guidelines. The type and dose remained unchanged during the study, unless safety concerns necessitated a dose reduction or discontinuation. Non‐study drug BIs were stopped by the time of randomization.

All participants provided informed, written consent. The clinical trial protocol was approved by the relevant health authorities and the appropriate local institutional review board/independent ethics committees. The study was conducted in accordance with the Declaration of Helsinki and the International Conference on Harmonization guidelines for good clinical practice.

### 2.2 Randomization

Participants were randomized 1:1 to self‐ or physician‐managed titration, using an interactive response system, stratified by HbA1c at screening (<8.5% vs ≥8.5% [<69 mmol/mol vs ≥69 mmol/mol]); previous use of insulin (yes vs no); and SU use at screening (yes vs no).

### 2.3 Interventions

After randomization, Gla‐300 was self‐administered subcutaneously once daily, with doses according to the European Union label, taking into account history of insulin use (Table S2). The time of daily administration was defined at the randomization visit by the participant and investigator, and maintained for the duration of the study, with occasional variation of ±3 h in injection time if necessary.

Paper diaries were dispensed to participants at each on‐site visit, and collected for review at each subsequent on‐site visit. Diaries contained instructions for managing glycaemic values according to each participant's allocated titration arm. Participants were trained at visits 1 and 2 (and re‐trained as necessary during the study) in how to complete these diaries to record information related to hypoglycaemic events, self‐monitored plasma glucose (SMPG) and insulin doses. Insulin dose was to be adjusted to a fasting SMPG target of 4.4 to 7.2 mmol/L as per 2015 ADA recommendations, while avoiding hypoglycaemia.^11^ During telephone call “visits”, participants were also asked about their last fasting SMPG values, insulin dose and any adverse event (including hypoglycaemic events) they had experienced. In both arms, the Gla‐300 dose was to be adjusted based on the median of the last three consecutive fasting SMPG values from the past 3 to 4 days and including the day of adjustment, according to the titration algorithm presented in Table S3. In the physician‐managed titration algorithm, doses were titrated at each visit according to study design (weekly for the first 8 weeks, bi‐weekly until week 12, and then monthly until week 24). In the self‐managed titration algorithm, the dose was titrated every 3 to 4 days. Participants in the self‐managed group were instructed on the use of the algorithm but made decisions regarding titration on their own. All participants received the same training on use of the injection device and titration algorithm prior to randomization, at the first and second site visit, and training was repeated as often as necessary until participants could demonstrate competent unaided use. It was expected that most titrations to reach the target fasting SMPG would occur during the first 12 weeks of treatment with Gla‐300, but titration to achieve target could continue until week 24.

The study consisted of a prespecified schedule of six on‐site and 10 telephone call visits. During visits, physician‐managed participants were instructed to self‐inject at a dose prescribed by the investigator. Self‐managed participants were instructed to continue the same titration approach, unless participant safety was at risk. Additional contacts (telephone, on‐site visit) between scheduled visits were available at the discretion of the investigator.

### 2.4 Rescue medication

Addition of a new anti‐hyperglycaemic drug or a dose increase of an existing anti‐hyperglycaemic non‐study drug, was performed based on the investigator's decision and local requirements. It was expected that the initiation of any rescue medication would be deferred until week 12 of treatment. Short‐term use (ie, 10 consecutive days) of short‐acting insulin (eg, due to acute illness or surgery) was not considered as rescue therapy.

### 2.5 Outcome measures

The primary outcome was non‐inferiority of glycaemic control, as assessed by the change from baseline to week 24 in HbA1c, achieved using the self‐ versus physician‐managed titration algorithm for Gla‐300. Key secondary outcomes included the percentage of participants reaching fasting SMPG target without experiencing hypoglycaemia that was severe and/or confirmed by a glucose measurement of <3.0 mmol/L (see below for a full definition); safety and tolerability, including hypoglycaemic events; and change in PROs according to total and subscale scores using the Diabetes Distress Scale (DDS)^24^ and the Diabetes Empowerment Scale (DES).^25^ The DDS evaluates patient distress attributable to diabetes burden; scores range from 1 (no problem) to 6 (serious problem), with scores of 2.0 to 2.9 indicating “moderate distress” and scores ≥3 indicating “high distress”. The DES evaluates patient confidence in self‐management of diabetes treatment; scores range from 1 to 5, with higher scores indicating greater confidence in self‐management. The minimum clinically important difference from baseline for each PRO score was defined as half the standard deviation of baseline scores, pooled across the titration groups. A full list of outcomes is presented in Table S5. Hypoglycaemia was assessed according to ADA definitions^26^; “confirmed and/or severe” hypoglycaemia was defined as any event that was documented symptomatic or asymptomatic with a plasma glucose measurement of ≤3.9 mmol/L or <3.0 mmol/L, and/or was severe (requiring third‐party assistance).

### 2.6 Statistical methods

A sample size of 592 was required to ensure that the two‐sided 95% confidence interval (CI) for the mean difference between the two titration approaches would not exceed 0.3% HbA1c, with 80% power assuming the SD is 1.3%, with a one‐sided test at the 2.5% significance level and that the true difference between the two dosing regimens of Gla‐300 is zero.

The primary efficacy analysis was performed in the intention‐to‐treat (ITT) population, comprising all randomized participants, irrespective of the titration arm at the time of the analysis. PROs were also analysed in the ITT population. The safety population was defined as all randomized participants who received at least one dose of Gla‐300, regardless of the amount of treatment administered.

The primary endpoint was analysed using a mixed‐effect model with a repeated measures approach, including fixed categorical effects of titration approach, visit, titration approach‐by‐visit interaction, randomization strata as well as continuous fixed covariates of baseline HbA1c and baseline HbA1c value‐by‐visit interaction. A stepwise closed testing approach was used for the primary efficacy variable to assess non‐inferiority and superiority. Non‐inferiority was demonstrated if the upper bound of the two‐sided 95% CI for the difference in the mean HbA1c change from baseline to week 24 between titration approaches was <0.3% HbA1c. If non‐inferiority was demonstrated, superiority was tested and demonstrated if the upper bound of the two‐sided 95% CI for the difference in the mean HbA1c change from baseline to week 24 between the two titration approaches was <0. Further details on statistical methods can be found in the Supporting Information.

The proportions of participants reaching fasting SMPG target and reaching fasting SMPG target without experiencing hypoglycaemia were analysed according to the mean of a log binomial regression model, adjusted on randomization strata of screening HbA1c, SU use, and previous use of insulin therapy. Safety endpoints were analysed descriptively.

## 3 RESULTS

### 3.1 Study population

Overall, 631 participants were randomized (ITT population) to self‐managed (314; 49.8%) or physician‐managed (317; 50.2%) titration (Figure S2). A similar proportion of participants in both groups completed the study; 307 (97.8%) and 311 (98.1%) in the self‐ and physician‐managed groups, respectively. One participant, in the physician‐managed group, discontinued the study because of poor treatment adherence. During the 24‐week study period, rescue medication was initiated in four (1.3%) and three (0.9%) participants from the patient‐ and physician‐managed groups, respectively.

Demographic and baseline characteristics were similar in the self‐ versus physician‐managed groups (Table 1). Across both groups, 50.6% of participants had received SUs as a non‐insulin anti‐hyperglycaemic treatment, 13.3% had received glucagon‐like peptide‐1 receptor agonists (GLP‐1RAs), and 13.0% had received sodium‐glucose co‐transporter‐2 (SGLT2) inhibitors (Table 1).

**Table 1:** Demographic characteristics

	Self‐managed n = 314	Physician‐managed n = 317	Total N = 631
Age, years	63.69 (8.84)	62.97 (9.00)	63.33 (8.92)
Sex, male, n (%)	158 (50.3)	159 (50.2)	317 (50.2)
Body weight, kg	89.08 (19.26)	88.36 (18.60)	88.72 (18.92)
BMI, kg/m^2^	31.68 (5.53)	31.75 (5.43)	31.71 (5.48)
HbA1c			
%	8.40 (0.89)	8.43 (0.92)	8.41 (0.90)
mmol/mol	68.3 (9.7)	68.6 (10.1)	68.4 (9.8)
Duration of T2DM, years	12.9 (7.2)	12.8 (6.9)	12.9 (7.0)
Prior non‐insulin antihyperglycaemic drugs, n (%)			
SUs	158 (50.3)	161 (50.8)	319 (50.6)
GLP‐1RAs	39 (12.4)	45 (14.2)	84 (13.3)
SGLT2 inhibitors	33 (10.5)	49 (15.5)	82 (13.0)
Prior number of non‐insulin antihyperglycaemic drugs, n (%)			
0	1 (0.3)	0 (0)	1 (0.2)
1	71 (22.6)	57 (18.0)	128 (20.3)
Metformin	49 (15.6)	40 (12.6)	89 (14.1)
SUs	10 (3.2)	7 (2.2)	17 (2.7)
2	169 (53.8)	181 (57.1)	350 (55.5)
Including metformin	157 (50.0)	173 (54.6)	330 (52.3)
>2	73 (23.2)	79 (24.9)	152 (24.1)
Previous insulin use, n (%)	195 (62.1)	195 (61.5)	390 (61.8)
Previous BI treatment, n (%)			
Number of participants	193	193	386
Insulin glargine	115 (59.6)	109 (56.5)	224 (58.0)
Insulin NPH	53 (27.5)	48 (24.9)	101 (26.2)
Insulin detemir	22 (11.4)	30 (15.5)	52 (13.5)
Other	3 (1.6)	6 (3.1)	9 (2.3)
Duration of prior BI use			
Number of participants	187	190	377
Years	3.5 (4.3)	3.6 (4.1)	3.5 (4.2)
Previous BI injection number, n (%)			
Number of participants	193	193	386
Once daily	180 (93.3)	168 (87.0)	348 (90.2)
Twice daily	12 (6.2)	24 (12.4)	36 (9.3)
Other	1 (0.5)	1 (0.5)	2 (0.5)

Abbreviations: BI, basal insulin; BMI, body mass index; GLP‐1RA, glucagon‐like peptide‐1 receptor agonist; HbA1c, glycated haemoglobin; SGLT2, sodium‐glucose co‐transporter‐2; SU, sulphonylurea; T2DM, type 2 diabetes.

Values are mean (SD) unless otherwise stated.

Overall, 62% of participants had received previous BI treatment, with a mean (SD) duration of 3.5 (4.2) years. In the self‐ and physician‐managed groups, 37.9% and 38.5% of participants, respectively, were insulin‐naïve.

### 3.2 Glycaemic control

**Figure 1:**
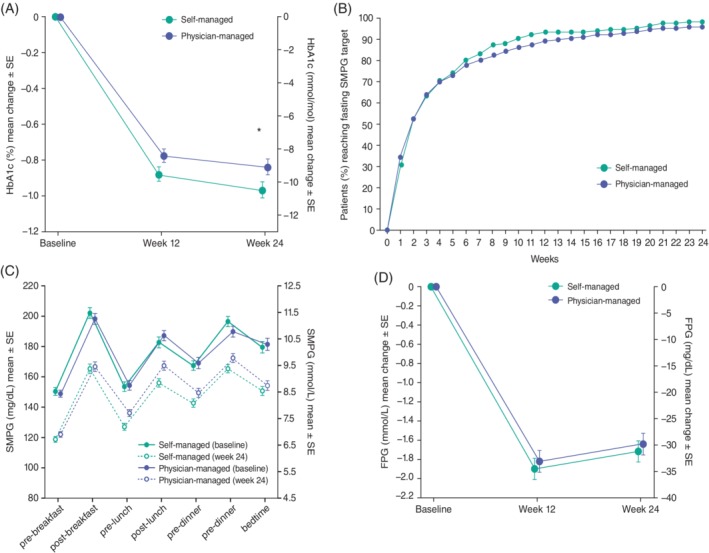
A, glycated haemoglobin (HbA1c) least squares (LS) means change between baseline and week 24. B, time to reach fasting self‐monitored plasma glucose (SMPG) target. C, seven‐point SMPG profiles between baseline and week 24. D, fasting plasma glucose (FPG) LS means change between baseline and week 24. Intention‐to‐treat population. *LS mean difference − 0.13 (95% CI −0.2619; −0.0004), *P* < 0.0001 (non‐inferiority); *P* = 0.0247 (superiority)

The least squares (LS) mean reduction in HbA1c from baseline to week 24 was 0.97% (10.6 mmol/mol) and 0.84% (9.2 mmol/mol) for self‐ and physician‐managed titration respectively, with an LS mean difference of −0.13% [95% confidence interval −0.2619 to −0.0004] (−1.4 mmol/mol [−2.863 to −0.004]) (Figure 1A). Non‐inferiority (*P* < 0.0001) and superiority (*P* = 0.0247) were demonstrated for self‐ versus physician‐managed titration. Mean HbA1c reduction from baseline to week 24 was greater in insulin‐naïve versus pre‐treated participants (−1.58 vs −0.57% [−17.3 vs –6.2 mmol/mol] in the self‐managed titration group; versus −1.48 vs −0.46% [16.2 vs −5.0 mmol/mol] in the physician‐managed titration group; Table S5).

After 24 weeks, more participants in the self‐ versus physician‐managed titration groups (30.4% vs 22.9%) achieved an HbA1c target <7.0% (<53 mmol/mol) (*P* = 0.0269; Table 2). Mean (SD) HbA1c levels reached 7.42 (0.96)% and 7.56 (0.88)% (57.6 [10.5] mmol/mol and 59.1 [9.6] mmol/mol) in the self‐ and physician‐managed groups, respectively.

**Table 2:** Achievement of glycaemic targets at week 24

	Self‐managed n = 314	Physician‐managed n = 317	RR (95% CI)	*P*
Proportion of patients achieving target HbA1c <7.0% (<53 mmol/mol)	30.43	22.91	1.33 (1.03 to 1.71)	0.0269
Proportion of patients achieving target fasting SMPG 4.4‐7.2 mmol/L	72.08	65.45	1.10 (0.99 to 1.22)	0.0615
Proportion of patients achieving target fasting SMPG 4.4‐7.2 mmol/L, without severe and/or confirmed (<3.0 mmol/L) hypoglycaemia	67.26	58.32	1.15 (1.02 to 1.30)	0.0187

Abbreviations: HbA1c, glycated haemoglobin; RR, relative risk; SMPG, self‐monitored plasma glucose.

At week 24, there was a similar proportion of participants who achieved target fasting SMPG (4.4‐7.2 mmol/L) in the self‐ and physician‐managed groups (72.1% and 65.5%; Table 2). Significantly more participants in the self‐ than in the physician‐managed group (67.3% and 58.3%) achieved fasting SMPG target without experiencing severe and/or confirmed (<3.0 mmol/L) hypoglycaemia (*P* = 0.0187; Table 2). By week 2, >50% of participants in both titration groups had achieved fasting SMPG target, while 98% and 95% of participants achieved fasting SMPG target by week 24 in the self‐ and physician‐managed titration groups, respectively (*P* = 0.2177; Figure 1B). The mean seven‐point SMPG profiles at baseline and week 24 were similar in the self‐ and physician‐managed titration groups (Figure 1C).

The fasting plasma glucose (FPG) LS mean change from baseline to week 24 was −1.72 mmol/L in the self‐managed titration group and −1.64 mmol/L in the physician‐managed titration group, resulting in an LS mean difference of −0.08 mmol/L (95% CI −0.39 to 0.24; Figure 1D).

### 3.3 Hypoglycaemia

During the 24‐week on‐treatment period, 36.2% of participants in the self‐managed titration group and 37.0% in the physician‐managed titration group experienced at least one episode of hypoglycaemia, of any definition (relative risk 0.98 [95% CI 0.80 to 1.20]; Table 3). Nocturnal hypoglycaemia (12:00‐5:59 am) of any type was reported by 8.0% of participants in the self‐managed titration group and 11.4% in the physician‐managed titration group (relative risk 0.70 [95% CI 0.43 to 1.13]). Severe hypoglycaemia was reported in two of 312 participants [0.6%] in the self‐managed titration group, and one of 316 participants [0.3%] in the physician‐managed titration group.

**Table 3:** Participants experiencing ≥1 emergent hypoglycaemia event during the 24‐week on‐treatment period^a^

	All hypoglycaemia		Nocturnal hypoglycaemia (12:00–5:59 am)	
	Self‐managed N = 312	Physician‐managed N = 316	Self‐managed N = 312	Physician‐managed N = 316
Any hypoglycaemia	113 (36.2)	117 (37.0)	25 (8.0)	36 (11.4)
RR (95% CI)	0.98 (0.80 to 1.20)		0.70 (0.43 to 1.13)	
Severe hypoglycaemia	2 (0.6)	1 (0.3)	0	1 (0.3)
RR (95% CI)	2.04 (0.19 to 22.24)		NE	
Documented symptomatic hypoglycaemia (≤3.9 mmol/L)	72 (23.1)	72 (22.8)	20 (6.4)	22 (7.0)
RR (95% CI)	1.02 (0.77 to 1.34)		0.90 (0.50 to 1.61)	
Documented symptomatic hypoglycaemia (<3.0 mmol/L)	20 (6.4)	20 (6.3)	4 (1.3)	5 (1.6)
RR (95% CI)	1.00 (0.55 to 1.82)		0.80 (0.22 to 2.94)	
Confirmed (≤3.9 mmol/L) and/or severe hypoglycaemia	104 (33.3)	108 (34.2)	21 (6.7)	32 (10.1)
RR (95% CI)	0.98 (0.79 to 1.21)		0.66 (0.39 to 1.11)	
Confirmed (<3.0 mmol/L) and/or severe hypoglycaemia	23 (7.4)	25 (7.9)	4 (1.3)	7 (2.2)
RR (95% CI)	0.92 (0.54 to 1.58)		0.58 (0.17 to 1.94)	

Abbreviations: NE, not evaluable; RR, risk ratio.

Safety population. Hypoglycaemia was defined according to American Diabetes Association criteria. N = Number of patients at risk; n (%) = number and percentage of patients with at least one hypoglycaemia event. The percentage is determined using the number of patients at risk within each treatment period as denominator.

a RR (95% CI) values refer to self‐ vs. physician‐managed titration groups.

The self‐ and physician‐managed titration groups had similar incidences of hypoglycaemia (≤3.9 mmol/L) in the documented symptomatic (23.1% and 22.8%), asymptomatic (17.0% and 20.9%), and confirmed and/or severe (33.3% and 34.2%) categories.

The incidence of confirmed (≤3.9 mmol/L and <3.0 mmol/L) and/or severe hypoglycaemia was similar between self‐ and physician‐managed titration, regardless of prior insulin use (Table S5).

### 3.4 Adverse events

Treatment‐emergent adverse events (TEAEs) were reported by 33.7% of participants in the self‐managed titration group, and 34.5% in the physician‐managed titration group during the 24‐week on‐treatment period (Table S5). Rates of serious TEAEs were similar in the self‐managed (3.2%) and the physician‐managed titration group (3.8%). No deaths occurred during the study. Only one participant in the physician‐managed titration group experienced a TEAE leading to permanent treatment discontinuation. The serious TEAE was acute heart failure, assessed as unrelated to treatment.

### 3.5 Insulin dose and body weight

There was a numerically higher mean daily insulin dose increase in the overall self‐managed versus physician‐managed group as well as in the insulin‐naïve and insulin pre‐treated subgroups of these titration approaches (Table 4). During the 24‐week on‐treatment period, a total of 3017 and 1713 dose adjustments were made in the self‐ and physician‐managed groups, respectively.

**Table 4:** Change from baseline in mean daily insulin dose

	Self‐managed			Physician‐managed		
	Insulin‐naïve n = 118	Insulin pre‐treated n = 194	Total N = 312	Insulin‐naïve n = 194	Insulin pre‐treated n = 122	Total N = 316
Baseline	17.43 (6.21)	28.18 (18.80)	24.11 (16.16)	16.32 (3.94)	31.66 (21.68)	25.74 (18.71)
Week 24	35.04 (20.98)	42.57 (27.76)	39.72 (25.63)	27.85 (14.38)	42.64 (26.89)	36.91 (23.95)
Change from baseline	17.72 (19.69)	14.24 (21.05)	15.56 (20.58)	11.59 (12.98)	10.95 (11.96)	11.20 (12.35)

Data are mean (SD) insulin dose, U.

The LS mean (SE) change in body weight from baseline to week 24, was +0.84 (0.17) kg in the self‐managed titration group and +0.50 (0.17) kg in the physician‐managed titration group (LS mean difference 0.34 [95% CI –0.14 to 0.82]).

### 3.6 Participant‐reported outcomes

The mean (SD) total DDS was 2.04 (0.89) and 2.06 (0.99) at baseline (indicating “moderate distress”) in the self‐ and physician‐managed groups. The mean change in the DDS total score from baseline to week 12 was −0.23 (0.70) and −0.23 (0.79) for the self‐ and physician‐managed groups, respectively. This reduction in DDS score from baseline was maintained at week 24 in both groups (−0.23 [0.76] and −0.17 [0.90]). The decrease in DDS total scores indicated small improvements in both groups, with an LS mean difference from baseline to week 24 of −0.07 (95% CI −0.19 to 0.04), for self‐ versus physician‐managed titration (Figure S3). The proportion of participants presenting “high distress” on the total DDS score at week 24 was 8.5% and 12.2% (relative risk 0.70 [95% CI 0.44 to 1.11]) in self‐ and physician‐managed titration groups, respectively. Similar changes in all DDS subscale scores were seen in both titration groups at week 24 (data not shown). In addition, for the emotional burden DDS subscale score at week 24, the proportion of participants presenting “high distress” was 13.1% and 19.7% (relative risk 0.66 [95% CI 0.47 to 0.95]) in the self‐ and physician‐managed groups, respectively. Moreover, the proportion of participants achieving the minimum clinically important difference (reduction to −0.57 or less from baseline in emotional burden DDS subscale score) at week 24 was 33.1% and 25.4% in the self‐ and physician‐managed groups, respectively (relative risk 1.30 [95% CI 1.02 to 1.66]).

The mean (SD) DES total scores at baseline were 4.00 (0.50) and 4.02 (0.51) in the self‐ and physician‐managed groups, indicating that both groups were reasonably confident in their diabetes self‐management. There was a mean increase in total DES scores from baseline to week 12 by 0.14 (0.43) and 0.06 (0.47) in the self‐ and physician‐managed groups, respectively. These increases in DES total score were maintained at week 24, with mean increases of 0.19 (0.45) and 0.12 (0.49) from baseline, and an LS mean difference (95% CI) of 0.07 (0.00 to 0.14) for self‐ versus physician‐managed titration, indicating that participants in both groups felt more confident in managing their diabetes (Figure S3). Similar improvements in all DES subscales were seen in both titration groups at week 24 (data not shown).

## 4 DISCUSSION

In the present study, self‐managed titration of Gla‐300 was both non‐inferior and superior to physician‐managed titration in terms of mean reduction in HbA1c from baseline to week 24. Slightly greater HbA1c reductions were observed in self‐ versus physician‐managed titration, regardless of previous insulin use, with more participants in the self‐managed group achieving the HbA1c target of <7.0% (<53 mmol/mol). Furthermore, a significantly higher proportion of participants in the self‐ versus physician‐managed groups achieved the fasting SMPG target without experiencing confirmed (<3.0 mmol/L) or severe hypoglycaemia.

The results from the present study are generally consistent with those from two previous studies. ATLAS^18^ and AT.LANTUS^16^, ^17^ were both 24‐week, multicentre, randomized, open‐label studies that demonstrated significant improvements in glycaemic control with self‐managed over physician‐managed titration of Gla‐100, in uncontrolled T2DM.^16^, ^17^, ^18^ There was no significant difference in the proportion of participants with self‐ versus physician‐managed titration achieving HbA1c <7.0% (<53 mmol/mol) without severe hypoglycaemia in ATLAS, or with severe hypoglycaemia in AT.LANTUS.^17^, ^18^ By contrast, target achievement without hypoglycaemia was improved with self‐ versus physician‐managed titration in the present Take Control study, although it should be noted that SMPG was used as the glycaemic target, rather than HbA1c, as in ATLAS and AT.LANTUS.

In AT.LANTUS and ATLAS, there was a higher risk of symptomatic hypoglycaemia with self ‐ vs physician‐managed titration.^17^, ^18^ Such results may cause physicians to be reluctant to encourage their patients to self‐titrate, in order to avoid hypoglycaemia. By contrast, the similar incidences of all definitions of hypoglycaemia reported in Take Control may reflect the improved pharmacokinetic properties of Gla‐300 versus Gla‐100,^20^, ^21^ which translated to lower incidence and rate of hypoglycaemia with Gla‐300 in previous randomized controlled trials and real‐world studies. Importantly, in Take Control there were very few hypoglycaemic events overall, with low and similar rates of TEAEs and serious TEAEs in both titration groups, suggesting that Gla‐300 would be better suited for self‐managed titration than Gla‐100.

At week 24, Gla‐300 doses were numerically higher with self‐ versus physician‐managed titration, consistent with the results with Gla‐100 in ATLAS and AT.LANTUS.^17^, ^18^ These higher doses may reflect the more frequent titration that is possible in the self‐managed algorithm. In Take Control, 1.8 times more dose adjustments were made in the self‐ versus physician‐managed group, which may explain the better glycaemic results, although the time taken to reach fasting SMPG target was similar in both groups. Nevertheless, as described above, the proportion of participants achieving HbA1c target was less than one‐third in both groups. While slightly higher proportions of participants achieve HbA1c targets in treat‐to‐target studies such as the EDITION programme,^22^ it appears that treatment intensification may be required in many patients. Given that >60% of participants achieved fasting SMPG targets but only 20% to 30% achieved HbA1c targets, additional treatment intensification with bolus insulin, addition of GLP‐1RA therapy, or other drugs that reduce postprandial glucose peaks may be beneficial in this population with relatively advanced T2DM (mean duration of diabetes of 13 years); however, the suboptimal HbA1c target achievement in Take Control may be related to the higher fasting SMPG target (4.4‐7.2 mmol/L) compared with the EDITION programme (4.4‐5.6 mmol/L).^22^

The PRO scores for both titration groups at baseline indicated moderate distress and reasonable confidence in self‐management. Total DDS and DES results improved similarly in both groups, while more individuals in the self‐managed group achieved a clinically meaningful improvement in the DDS emotional burden subscale score, and fewer individuals reported a high emotional burden compared to the physician‐managed group. These results indicate that, by taking responsibility for titrating insulin, patients did not experience more distress related to diabetes, had a tendency towards less emotional burden, and had similar confidence in self‐management of their diabetes versus physician management. In the ATLAS study, there was no difference in the treatment satisfaction or quality‐of‐life outcomes between self‐ or physician‐managed titration of Gla‐100. It remains to be evaluated whether the PRO improvements observed with Gla‐300 in the present study translate to benefits in treatment satisfaction, overall quality of life, and treatment adherence and persistence in real‐life settings.

The study benefitted from the randomized trial design with a sufficiently large sample size to detect differences in the primary endpoint. Furthermore, the interactions between participants and research staff mimicked real‐life practice, with the same number of predefined visits in both groups. Participants were recruited from 79 centres in 10 European countries, with characteristics that were representative of the European T2DM population (mean age of 63 years, duration of diabetes of 13 years, and HbA1c 8.4% [68 mmol/mol] at baseline). There was good retention of participants in both titration groups, with ~98% completing the study. The study also benefitted from the simple titration algorithm and close follow‐up during the first 8 weeks. Given that barriers to effective insulin therapy include patient frustration at the time taken to achieve goals, empowering patients to titrate frequently using this simple titration algorithm is an important consideration for applying this approach in real‐life clinical practice. Other care approaches such as the Stepping Up model, whereby patients and practice nurses have an enhanced role in therapeutic decisions, have also shown improvements in insulin initiation rates and glycaemic control compared with standard practice^27^; therefore, involving patients more in the management of T2DM may provide benefits not just in treatment persistence and clinical outcomes, but also in the effective use of healthcare resources, and has been emphasized as a key consideration in the latest ADA/EASD consensus report.^15^

The study was limited by the lack of ability to blind participants to their treatment; however, if anything, this may be more likely to bias perception in the self‐managed titration group. The weekly contacts during the study may not reflect the level of interaction that patients would receive in routine practice. Furthermore, since the self‐managed algorithm allowed more frequent titration than the physician‐managed algorithm, one cannot directly conclude whether the results were driven by the patient or the titration regimen. However, it does indicate that the more frequent dose adjustments in the self‐managed group translated to better glycaemic control, with more participants achieving glycaemic targets without hypoglycaemia, and comparable or lower levels of distress than in the physician‐managed group, with a low overall risk of hypoglycaemia. It is also important to note that although participant characteristics at baseline were similar in the two titration groups, the results may not be generalizable to all patients with diabetes because other factors such as education level may impact the success of self‐titration. The results do show, however, that self‐titration is effective if performed appropriately.

The results of the Take Control study show that Gla‐300, a second‐generation BI analogue, with its prolonged, more stable and reproducible pharmacokinetic/pharmacodynamic profile,^20^ appears well suited to self‐managed titration. Encouragement and support of self‐titration in appropriate target groups may help to improve outcomes and provide a sense of empowerment in people with T2DM, without increasing distress.

## CONFLICT OF INTEREST

D. R.‐J. has received honoraria, teaching and research grants from AstraZeneca, Boehringer Ingelheim, Eli Lilly, GlaxoSmithKline, Novartis, Novo Nordisk, Pfizer and Sanofi‐Aventis. A.D., L.P. M.B. and A.R.C. are employees/shareholders of Sanofi. E.D. has received unrestricted research support from AstraZeneca, Novo Nordisk, Sanofi, Pfizer and Roche and has received consulting fees and/or honoraria for membership on advisory boards from AstraZeneca, Novo Nordisk, Lilly, Sanofi, GlaxoSmithKline, Pfizer, Almirall, Novartis, Abbott Laboratories, Esteve and Merck Sharp & Dohme. G.D. has received honoraria for lectures/advisory boards and research support (through the University of Athens) from Abbott, AstraZeneca, Lilly, ELPEN, MSD, Novartis, Novo Nordisk, Sanofi and Vianex. H.A.F. has received honoraria for advisory boards with Novo Nordisk, Sanofi‐Aventis, Lilly and Abbott. B.S. has acted as consultant, advisory board member or speaker for Sanofi‐Aventis, Novo Nordisk, Lilly, Merck Sharp & Dohme, Boehringer Ingelheim, AstraZeneca, DEXCOM and Ypsomed. K.S. has received honoraria from Sanofi Aventis, Novo Nordisk, Servier, AstraZeneca, Eli Lilly, MSD, Boehringer Ingelheim, Krka and research support from AstraZeneca, Pfizer, Sanofi Aventis, Novo Nordisk and Amgen. M.D. has acted as consultant, advisory board member and speaker for Novo Nordisk, Sanofi‐Aventis, Lilly, Merck Sharp & Dohme, Boehringer Ingelheim, AstraZeneca and Janssen, an advisory board member for Servier and as a speaker for Mitsubishi Tanabe Pharma Corporation and Takeda Pharmaceuticals International Inc. She has received grants in support of investigator and investigator‐initiated trials from Novo Nordisk, Sanofi‐Aventis, Lilly, Boehringer Ingelheim and Janssen.

## AUTHOR CONTRIBUTIONS

D.R.‐J. contributed to the original design of the trial, participated as an investigator, provided comments and input to all drafts of the manuscript and interpretation of the results. A.D. provided comments and input to all drafts of the manuscript and interpretation of the results. E.D. participated as an investigator, provided comments and input to all drafts of the manuscript and interpretation of the results. G.D. participated as an investigator, provided comments and input to all drafts of the manuscript and interpretation of the results. H.A.F. participated as an investigator, provided comments and input to all drafts of the manuscript and interpretation of the results. L.P. was esponsible for medical oversight during the trial, provided comments and input to all drafts of the manuscript and interpretation of the results. B.S. participated as an investigator, provided comments and input to all drafts of the manuscript and interpretation of the results. K.S. participated as an investigator, provided comments and input to all drafts of the manuscript and interpretation of the results. M.B. wrote the study outline, provided comments and input to all drafts of the manuscript and interpretation of the results. A.R.C. provided the analysis and critical interpretation of the PRO data. M.D. contributed to the original design of the trial, participated as an investigator, provided comments and input to all drafts of the manuscript and interpretation of the results.

## DATA SHARING

Qualified researchers may request access to patient‐level data and related study documents including the clinical study report, study protocol with any amendments, blank case report form, statistical analysis plan, and dataset specifications. Patient‐level data will be anonymized and study documents will be redacted to protect the privacy of trial participants. Further details on Sanofi's data sharing criteria, eligible studies, and process for requesting access can be found at: https:// www.clinicalstudydatarequest.com.

## Supporting information


**Table S1** Inclusion and exclusion criteria
**Table S2:** Gla‐300 starting doses
**Table S3:** Titration algorithm
**Table S4.** Study objectives
**Table S5.** HbA_1c_ reduction and hypoglycaemia incidence according to prior insulin use
**Table S6.** Adverse events during 24‐week on‐treatment period

**Figure S1** Take Control study design

**Figure S2** Participant flow diagram

**Figure S3** Change in patient reported outcomes A) Diabetes Distress Scale (DDS) total scores B) Diabetes Empowerment Scale (DES) total scores
