# Supplementary material for: Take Control: A randomized trial evaluating the efficacy and safety of self‐ versus physician‐managed titration of insulin glargine 300 U/mL in patients with uncontrolled type 2 diabetes
Source: Diabetes Obes Metab. 2019 Apr 30;21(7):1615–24. doi: 10.1111/dom.13697 (PMC6767413; doi:10.1111/dom.13697)

**Supplementary methods**

**Statistical methods**

A sample size of 592 subjects (296 per group) was required to ensure that the two-sided 95% confidence interval (CI) for the mean difference between the two titration modalities would not exceed 0.3 % HbA_1c_, with 80% power assuming the standard deviation (SD) is 1.3 %, with a 1-sided test at the 2.5% significance level and that the true difference between the two dosing regimens of Gla-300 is zero.

The primary endpoint was analyzed using a mixed-effect model with repeated measures approach, under the missing at random framework carried out via SAS PROC MIXED using an adequate contrast at visit 15 (week 24). The model included fixed categorical effects of titration modalities arm, visit (week 12 and 24), titration modalities-by-visit interaction, randomization strata of SU used at screening and previous use of insulin as well as continuous fixed covariates of baseline HbA_1c_ and baseline HbA_1c_ value-by-visit interaction. This model provided baseline adjusted least squares (LS) means estimates at week 24 for both titration modalities, as well as the differences of these estimates, with their corresponding standard errors (SEs) and 95% CIs. A stepwise closed testing approach was used for the primary efficacy variable to assess non-inferiority and superiority. Non-inferiority was demonstrated if the upper bound of the two-sided 95% CI for the difference in the mean HbA_1c_ change from baseline to week 24 between self- and physician-managed titration was <0.3 % HbA_1c_. If non-inferiority was demonstrated, superiority was tested and demonstrated if the upper bound of the two-sided 95% CI for the difference in the mean HbA_1c_ change from baseline to week 24 between the two titration modalities was <0.

The proportion of participants reaching fasting SMPG target and reaching fasting SMPG target without experiencing severe and/or confirmed (<3.0 mmol/L) hypoglycaemia was analyzed by the mean of a log binomial regression model adjusted on randomization strata of screening HbA_1c_, SU use, and previous use of insulin therapy. Safety endpoints were analyzed descriptively.

**Supplementary Table 1. Inclusion and exclusion criteria**

| **Inclusion criteria** |
| --- |
| Patients with T2DM diagnosed for ≥1 year at the time of screening, treated with  ≥1 non-insulin antihyperglycaemic drug(s) with or without a basal insulin, for  ≥6 months |
| Signed written informed consent |
| **Exclusion criteria** |
| Age <18 years |
| HbA_1c_ at screening:   - <7.0 % or >10.0 % for patients taking basal insulin - <7.5 % or >11.0 % for insulin-naïve patients |
| Patient not willing to self-manage titration algorithm (including self-injection and SMPG) |
| T1DM |
| Insulin-pretreated patients not on a stable BI regimen in the last 12 weeks prior to screening; the insulin dose should be stable (±20%) for ≥8 weeks prior to screening |
| Change in dose of existing, or initiating new, non-insulin antihyperglycaemic drugs in the last 12 weeks prior to screening |
| Treatment with insulin other than BI: premixed insulin, rapid insulin, fast acting insulin analogues in the last six months prior to screening (unless used for ≤10 days in relation to hospitalization or an acute illness) |
| Use of systematic glucocorticoids (excluding topical application or inhaled forms) for ≥2 weeks within an 8-week period prior to screening |
| History of hypoglycaemia unawareness |
| Any clinically significant abnormality identified through physical examination, laboratory test, or vital signs at screening, or any condition (including known substance/alcohol abuse or psychiatric disorder) that, in the opinion of the investigator/sub-investigator, would make implementation of the protocol or interpretation of the study results difficult or would precluded the safe participation of the subject |
| Use of any investigational drug within one month or five half-lives (whichever is longer) prior to screening |
| Patients included (or planned to be included during the study duration) in the  Gla-300 customized patient solution or any other patient support program |
| Pregnant/breastfeeding women. Women of childbearing potential, not protected by highly effective contraception and/or who are unwilling or unable to be tested for pregnancy |
| Known hypersensitivity/intolerance to Gla-300 or any of its excipients |

**Supplementary Table 2: Gla-300 starting doses**

| **Prior BI use** | **Starting daily dose of Gla-300** |
| --- | --- |
| Insulin naïve | 0.2 U/kg |
| Switching from once daily BI | Unit to unit basis, according to previous BI dose |
| Switching from twice daily BI | 80% of total daily dose of previous BI |

**Supplementary Table 3: Titration algorithm**

| **Median* of last 3 consecutive fasting SMPGs from last 3–4 days in the range of:** | **Dose adjustment HOE901-U300 (unit/day)** |
| --- | --- |
| >7.2 mmol/L | +3 |
| Glycaemic target: 4.4 to 7.2 mmol/L, both inclusive | No change |
| <4.4 mmol/L or occurrence of ≥2 symptomatic or 1 severe hypoglycaemia episode in the preceding week | −3 or at the discretion of the investigator |

*Median refers to intermediate SMPG value (the value between the lowest and the highest SMPG values when the values are ranked in a growing order)

**Supplementary Table 4. Study objectives**

| **Primary objective** |
| --- |
| To demonstrate non-inferiority in terms of glycaemic control, measured as change from baseline to week 24 in HbA_1c_, of a patient- versus a physician-managed titration algorithm, for the treatment with Gla-300, in patients with inadequately controlled T2DM |
| **Secondary objective** |
| To evaluate the efficacy, safety and quality of life of the two titration approaches in terms of: |
| Percentage of patients reaching fasting self-monitored SMPG target |
| Hypoglycaemic events |
| Change in HbA_1c_ from baseline across subgroups of baseline HbA_1c_ categories (<8 %, ≥8 to <9 %, ≥9 %). |
| Percentage of patients requiring rescue therapy |
| Safety and tolerability |
| Change in patient-reported outcome (PRO) instruments (Diabetes Distress Scale and Diabetes Empowerment Scale) |

**Supplementary Table 5. HbA_1c_ reduction and hypoglycaemia incidence according to prior insulin use**

|  | **Insulin pre-treated**  **(N=390)** | | **Insulin naïve (N=241)** | |
| --- | --- | --- | --- | --- |
|  | **Self-led titration**  **(N=195)** | **Physician-led**  **(N=195)** | **Self-led titration**  **(N=119)** | **Physician-led**  **(N=122)** |
| Mean baseline HbA_1c_, % (SD) | 8.15 (0.78) | 8.16 (0.72) | 8.80 (0.90) | 8.83 (1.04) |
| Mean week 24 HbA_1c_, % (SD) | 7.56 (1.00) | 7.69 (0.90) | 7.20 (0.85) | 7.35 (0.80) |
| HbA_1c_ mean change from baseline to week 24, % (SD) | −0.57 (0.88) | −0.46 (0.87) | −1.58 (1.04) | −1.48 (1.02) |
| HbA_1c_ LS mean difference self- vs physician-managed titration, % [95% CI] | −0.13 [−0.30 to 0.04] | | −0.14 [−0.34 to 0.06] | |
| Incidence of confirmed (≤3.9 mmol/L) or severe hypoglycaemia at any time of day (24 h), n (%)  Risk ratio self- vs physician-managed titration [95% CI] | 65 (33.5) | 75 (38.7) | 39 (33.1) | 33 (27.0) |
|  | 0.89 [0.69 to 1.15] | | 1.21 [0.82 to 1.77] | |
| Incidence of confirmed (<3.0 mmol/L) or severe hypoglycaemia at any time of day (24 h), n (%)  Risk ratio self- vs physician-managed titration [95% CI] | 14 (7.2) | 21 (10.8) | 9 (7.6) | 4 (3.3) |
|  | 0.67 [0.35 to 1.27] | | 2.32 [0.74 to 7.31] | |

**Supplementary Table 6. Adverse events during 24-week on-treatment period**

| **Event** | **Self-managed titration**  **(N=312)** | **Physician-managed titration**  **(N=316)** |
| --- | --- | --- |
| Any TEAE | 105 (33.7) | 109 (34.5) |
| Any serious TEAE | 10 (3.2) | 12 (3.8) |
| Any TEAE leading to death | 0 | 0 |
| Any TEAE leading to permanent treatment discontinuation | 0 | 1 (0.3) |
| Any TEAE related to Gla-300 | 7 (2.2) | 4 (1.3) |
| Any TEAE related to concomitant antidiabetic drug (not Gla-300) | 3 (1.0) | 3 (0.9) |
| Any TEAE related to study procedures or devices | 0 | 0 |
| Any AESI | 1 (0.3) | 0 |

n (%) = number and percentage of patients with at least one TEAE. 24-week on-treatment period is defined as the time from the first dose of IMP up to 2 days after the last dose of IMP, regardless of the introduction of rescue therapy.

TEAE, treatment emergent adverse events IMP; AESI, adverse event of special interest (ALT increase, overdose, pregnancy)

**Supplementary Figure 1: Take Control study design**


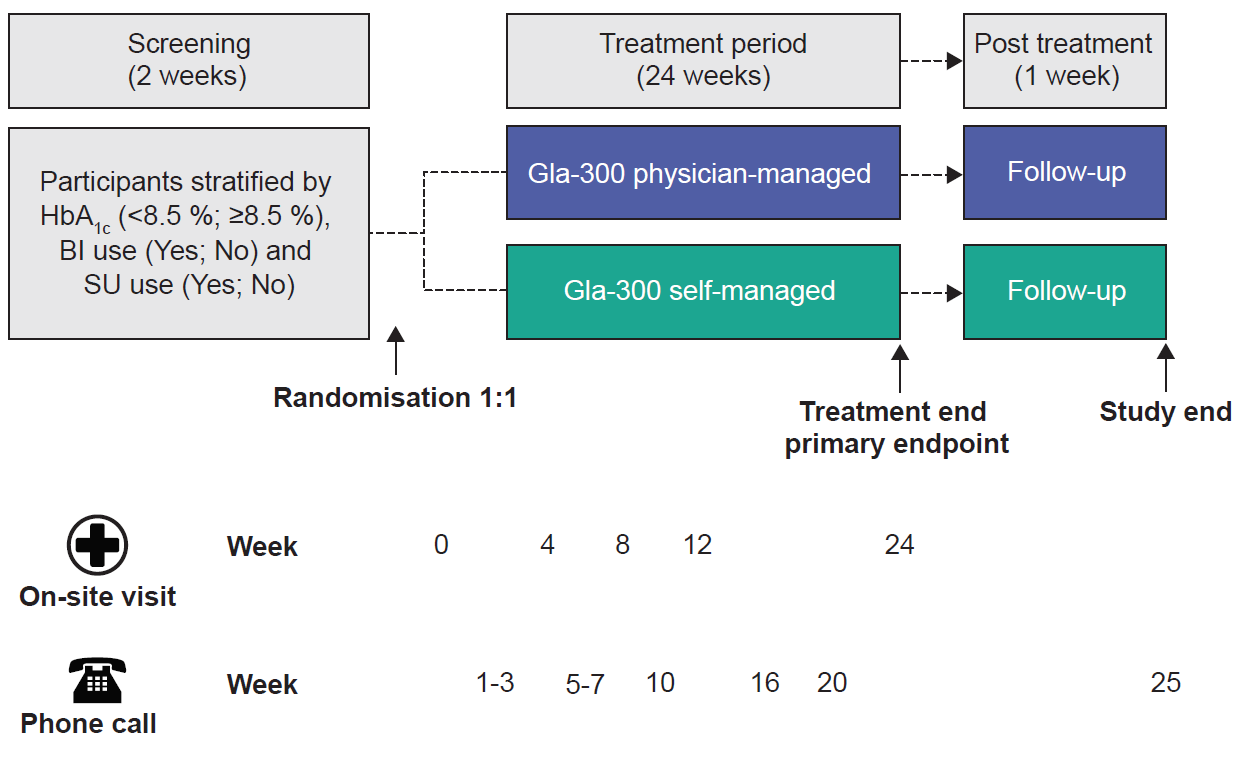


BI, basal insulin; HbA_1c_, glycated hemoglobin; SU, sulfonylurea

**Supplementary Figure 2. Participant flow diagram**


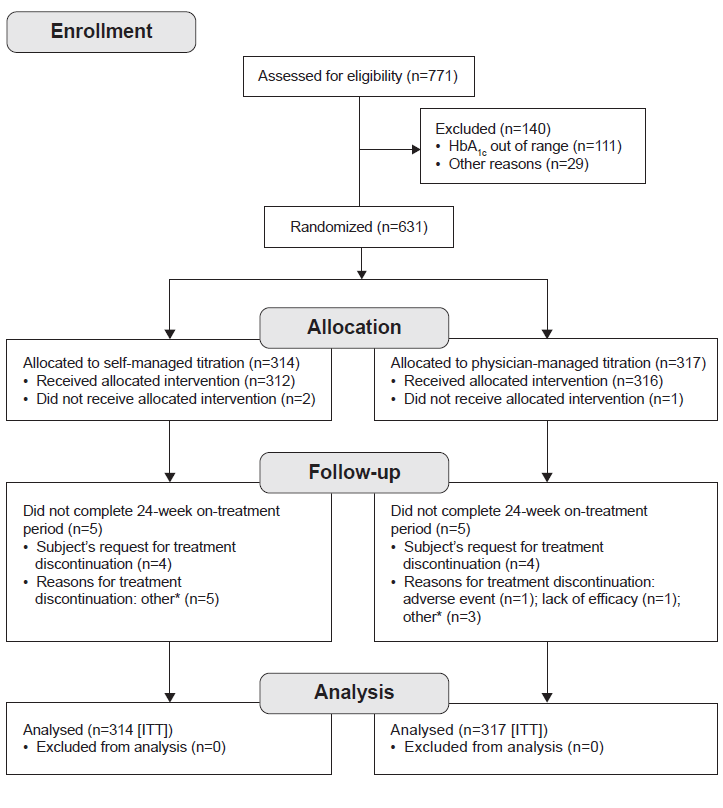


*Other reasons for treatment discontinuation include leaving the country, patient refusal to continue (not safety related), patient visited a different physician and treatment was changed

**Supplementary Figure 3. Change in patient reported outcomes A) Diabetes Distress Scale (DDS) total scores B) Diabetes Empowerment Scale (DES) total scores**


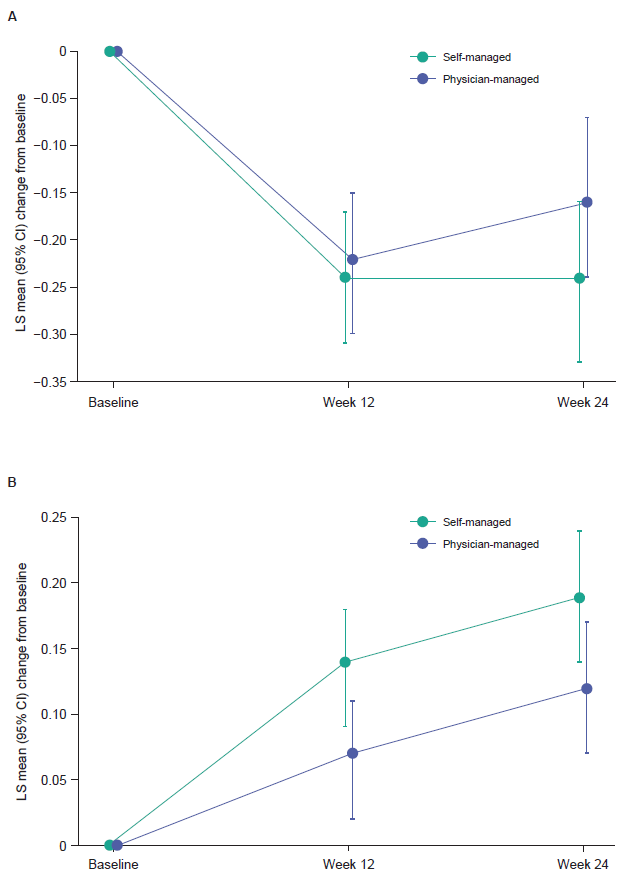

Supplement: Supplementary file 1 — Table S1 Inclusion and exclusion criteria Table S2: Gla‐300 starting doses Table S3: Titration algorithm Table S4. Study objectives Table S5. HbA1c reduction and hypoglycaemia incidence according to prior insulin use Table S6. Adverse events during 24‐week on‐treatment period [file DOM-21-1615-s001.docx]
